# Sex-Specific Associations Between Low Muscle Mass and Glucose Fluctuations in Patients With Type 2 Diabetes Mellitus

**DOI:** 10.3389/fendo.2022.913207

**Published:** 2022-07-13

**Authors:** Xiulin Shi, Wenjuan Liu, Lulu Zhang, Fangsen Xiao, Peiying Huang, Bing Yan, Yiping Zhang, Weijuan Su, Qiuhui Jiang, Mingzhu Lin, Wei Liu, Xuejun Li

**Affiliations:** ^1^ Department of Endocrinology and Diabetes, Xiamen Diabetes Institute, Fujian Province Key Laboratory of Translational Research for Diabetes, The First Affiliated Hospital of Xiamen University, School of Medicine, Xiamen University, Xiamen, China; ^2^ The School of Clinical Medicine, Fujian Medical University, Fouzhou, China; ^3^ Department of Endocrine, Zhangzhou Hospital of Traditional Chinese Medicine, Zhangzhou, China

**Keywords:** low muscle mass, glucose fluctuations, sex-specific, type 2 diabetes mellitus, continuous subcutaneous insulin infusion

## Abstract

**Objective:**

Studies have shown that sex differences in lean mass, concentrations of sex hormones, and lifestyles influence cle health and glucose metabolism. We evaluated the sex-specific association between low muscle mass and glucose fluctuations in hospitalized patients with type 2 diabetes mellitus (T2DM) receiving continuous subcutaneous insulin infusion (CSII) therapy.

**Methods:**

A total of 1084 participants were included. Body composition was determined by dual-energy X-ray absorptiometry. Intraday blood glucose fluctuation was estimated by the Largest amplitude of glycemic excursions (LAGE) and standard deviation of blood glucose (SDBG).

**Results:**

The prevalence of low muscle mass was higher in males than in females (*p*<0.001). There was a significant sex-specific interaction between the status of low muscle mass and glucose fluctuations (LAGE and SDBG) (*p* for interaction=0.025 and 0.036 for SDBG and LAGE, respectively). Among males, low muscle mass was significantly associated with a higher LAGE and SDBG (difference in LAGE: 2.26 [95% CI: 1.01 to 3.51], *p* < 0.001; difference in SDBG: 0.45 [95% CI: 0.25 to 0.65], *p* < 0.001) after adjustment for HbA1c, diabetes duration, hyperlipidemia, diabetic peripheral neuropathy, diabetic nephropathy, and cardiovascular disease. These associations remained significant after further adjustment for age and C-peptide. Among females, low muscle mass was not associated with LAGE or SDBG after adjustment for all covariates.

**Conclusion:**

The prevalence of low muscle mass was higher in males than in females. Low muscle mass was significantly associated with higher LAGE and SDBG among males, but not females.

## Introduction

Diabetes is a major public health challenge in the world due to its high and increasing prevalence and related risk of chronic complications and mortality. Accumulating evidence indicates that glucose fluctuations are more harmful in the occurrence and development of diabetic chronic complications compared to constant hyperglycemia (1–3). Generalized and progressive skeletal muscle function disorder is the definition of sarcopenia, which includes progressive loss of muscle mass and function leading to adverse outcomes such as functional decline, frailty, falls, and mortality (4). The prevalence of sarcopenia is significantly higher in type 2 diabetes mellitus (T2DM) than in non-diabetic individuals (5–7). Sarcopenia has been implicated as both a cause and consequence of T2DM (8, 9). The progressive loss of the skeletal muscle might lead to diminished insulin-mediated glucose disposal and exacerbated insulin resistance, resulting in severe glucose abnormalities (10). It has been demonstrated that not only the glycosylated hemoglobin A1c (HbA1c) level but also glucose fluctuations were significantly related to sarcopenia (11).

Lean mass, body fat composition and distribution, hormone concentrations, and lifestyles showed a difference between males and females, which influenced muscle health and glucose metabolism (12). Lean mass, which is generally greater in men, may play an important role in mediating the regulation of glucose metabolism by skeletal muscle. However, to the best of our knowledge, no study focusing on the potential impact of sex differences on the relationship between low muscle mass and glucose fluctuations has been reported.

In this study, we aimed to assess the sex-specific relationship between low muscle mass and glucose fluctuations in hospitalized patients with T2DM undergoing continuous subcutaneous insulin infusion (CSII) treatments.

## Materials and Methods

### Study Design and Participants

The study was performed following the rules of the Declaration of Helsinki, and the protocol was approved by the ethics committee of the First Affiliated Hospital of Xiamen University. All participants provided written informed consent before participating in the study. We included 1084 hospitalized patients for hyperglycemia in the Department of Endocrinology and Diabetes, First Affiliated Hospital, Xiamen University, Xiamen, China from 2017 to 2019. The included criteria for patients were as follows: patients aged 35 years or older with T2DM which was defined as having either fasting plasma glucose (FPG)≥7.0mmol/l or 2-h PG ≥11.1 mmol/l according to the World Health Organization definition. The exclusion criteria were as follows (1): serious health conditions, such as diabetic ketoacidosis, severe hepatic insufficiency, moderate to severe renal insufficiency, cardiac insufficiency, or stroke affected daily activities (2); cognitive disability or an inability to cooperate with the examination (3); pregnant or contemplating pregnancy.

All patients were managed according to established protocols for performing CSII with a length of 7 days, glucose monitoring, and dual-energy X-ray absorptiometry. Diabetes-associated chronic complications were evaluated for the coexistence of neuropathy, retinopathy, and nephropathy.

### Glucose Control

On the first day of hospitalization, oral hypoglycemic agents used were stopped, then Humalog rapid-acting insulin (insulin lispro; Eli Lilly, Indianapolis, IN, USA) with the insulin pump (MiniMed Paradigm 722, Medtronic, Northridge, CA, USA) was used among all patients used. The initial insulin dosage was 0.7 unit × body weight (kg) and total daily doses were divided into 50% of basal and 50% of bolus injections. The dawn phenomenon and nocturnal hypoglycemia were taken into account, and the basal rate was fixed depending on the period: basal insulin dose/24 × 0.8 between 2200 and 0300 hours; basal insulin dose/24 × 1.2 between 0300 and 0700 hours; basal insulin dose/24 × 1.0 between 0700 and 2200 hours. The basal and bolus doses of insulin infusion were tailored every 2 days by one doctor by 0.3 unit/h and 3 units, respectively, according to the capillary blood glucose (BG) level to achieve the glycemic target (fasting BG<7.0 mmol/L and average postprandial BG <10.0 mmol/L). All patients received the same education for lifestyle management, and they were fed by the hospital nutrition canteen during the hospitalization.

BG was monitored 7 times per day (before and 2 h after each meal and at bedtime) by a trained nurse using a unified glucometer (Johnson & Johnson, New Brunswick, NJ, USA). Hypoglycemia was defined as a glucose level less than 3.9 mmol/L, and the presence or absence of hypoglycemic symptoms was recorded at every BG measurement point.

### Date Collection

Data were collected from electronic health records in the hospital. The clinical condition and medical history of all participants were obtained, including smoking, alcohol consumption habit, medical history (cardiovascular disease, hypertension, diabetic neuropathy, diabetic retinopathy, and diabetic nephropathy), previous hospitalizations, as well as regular antidiabetic drugs, *etc.* Blood and urine samples were taken the day following admission after overnight fasting. The following biochemical parameters were obtained: HbA1c, C-peptide, triglyceride (TG), total cholesterol (TC), high-density lipoprotein cholesterol (HDL-c), and low-density lipoprotein cholesterol (LDL-c), serum albumin, alanine aminotransferase (ALT), aspartate aminotransferase (AST), and urinary albumin.

### Anthropometric and Body Composition Measurements

Height and weight were measured by trained nurses according to the standard protocol and body mass index (BMI) was calculated as weight (kilograms) divided by height (meters) squared. BMI was further categorized into four groups:<18.5, 18.5–23.9, 24.0–27.9, and ≥28.0 kg/m^2^, according to the Chinese BMI cut-offs (13). Blood pressure was measured with a standard electronic sphygmomanometer on the right arm 5 minutes after sitting for rest. Body composition was determined by dual-energy X-ray absorptiometry (HOLOGIC Discovery A) on the first day of the hospitalization. The appendicular skeletal muscle mass index (ASMI) was calculated by dividing the appendicular skeletal muscle mass by the height squared (kg/m^2^).

### Evaluation of Low Muscle Mass, Glucose Fluctuation, and Pancreatic β-Cell Function

The Asian specific cutoff point for diagnosis of low muscle mass was according to the recommendation of the Asian Working Group for Sarcopenia (AWGS) in 2014 (14). Participants with an ASMI less than 7.0 Kg/m^2^ for men or 5.4 Kg/m^2^ for women were considered to have low muscle mass. The largest amplitude of glycemic excursions (LAGE), mean blood glucose (MBG), the standard deviation of blood glucose (SDBG), postprandial glucose excursion (PPGE) and were indicators for estimating intraday blood glucose fluctuations (3, 14). The MBG was the average glucose for 7 days. SDBG was calculated as the square root of the variance of the daily blood glucose for a whole day during the 7 days of hospitalization, respectively (15). LAGE was determined based on the mean of the diurnal range from minimum glucose levels to maximum glucose levels of BG for 7 days. PPGE was calculated based on the mean of the difference between pre-prandial and 2-h postprandial glucose. C-peptide was a more accurate marker of endogenous insulin secretion than insulin (16, 17). Fasting plasma C-peptide was measured to represent an index of pancreatic β-cell function.

### Statistical Analysis

To assess our hypothesis that the sex-specific association between low muscle mass and glucose fluctuations in hospitalized patients with T2DM with CSII therapy, several analyses were performed. Data were summarized using frequencies and counts for categorical variables and means and standard deviations for continuous variables. Student’s *t*-tests or the Mann-Whitney *U* test for continuous variables and Chi-square (*χ* ^2^) test for categorical variables were performed to compare the difference in baseline characteristics between diabetic patients with low muscle mass and diabetic patients with non-low muscle mass. An *a priori* sex-specific association between glucose fluctuations and sarcopenia was examined. We used multiple linear regression models to examine the association between low muscle mass and glucose fluctuations (SDBG and LAGE), after adjustment for HbA1c, diabetes duration, hyperlipidemia, diabetic peripheral neuropathy, diabetic nephropathy, and cardiovascular disease in Model 1. We further adjusted for age in Model 2. To further explore whether the relationship was independent of C-peptide, we additionally controlled for diabetes duration in Model 3.

Potential modification effects were assessed through a stratified analysis by the following factors: age (<65 or ≥65), BMI (<18.5, 18.5-23.9, 24.0-27.9, ≥28.0), diabetes duration (<5, 5-9.9, ≥10), diabetic peripheral nephropathy (yes or no), diabetic neuropathy (yes or no), and cardiovascular disease (yes or no). We evaluated the potential effect of modification by modeling the cross-product term of the stratifying variable with low muscle mass.

Two-tailed *p*-value < 0.05 was considered statistically significant. The data analysis for this article was conducted using SAS version 9.4.

## Results

**Figure 1:**
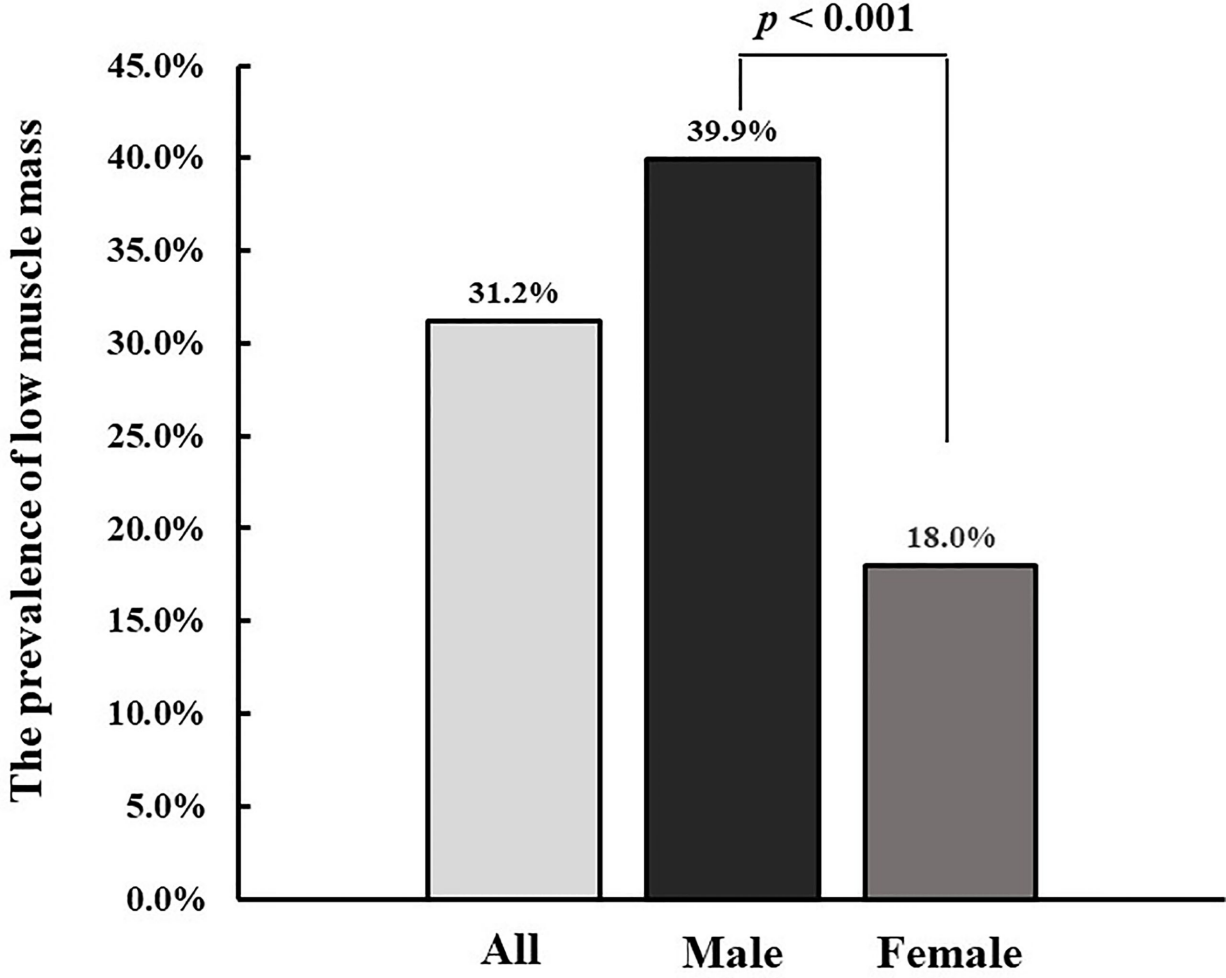
The prevalence of low muscle mass among all patients, male and female, male vs female:

Overall, the prevalence of low muscle mass was 31.2% in all diabetic participants. The prevalence of low muscle mass was higher in males than in females (39.9% vs 18.0%, *p*<0.001) (**Figure 1**). The characteristics of participants were shown in **Table 1** according to the status of low muscle mass and sex of patients. For males, participants with low muscle mass were older, and more likely to have a lower BMI, lower SBP and DBP, longer diabetes duration, higher LDL-c, LAGE, MBG, SDBG, and PPGE, lower TG and C-peptide, and higher prevalence of diabetic neuropathy and hypoglycemia compared with patients without low muscle mass. For females, BMI, SBP, DBP, C-peptide, TG, ALT, and AST were lower in patients with low muscle mass than in those without low muscle mass.

**Table 1:** Characteristics of the T2DM participants in the Low muscle mass group and non- Low muscle mass group stratified by sex.

	Female			Male		
	Non- Low muscle mass	Low muscle mass	*p* value	Non- Low muscle mass	Low muscle mass	*p* value
N	355	78		391	260	
Age, mean (SD), y	56.1 (11.5)	57.4 (14.5)	0.369	50.7 (11.7)	53.2 (13.6)	0.014
BMI, mean (SD), kg/m2	25.0 (3.9)	19.8 (2.1)	<0.001	26.2 (3.2)	21.6 (2.5)	<0.001
Systolic BP, mean (SD), mmHg	134.4 (20.7)	123.6 (19.0)	<0.001	130.3 (16.9)	126.4 (18.0)	0.006
Diastolic BP, mean (SD), mmHg	80.6 (10.3)	73.7 (9.0)	<0.001	81.9 (9.8)	78.1 (10.6)	<0.001
Diabetes duration, mean (SD), y	8.5 (2.3)	8.5 (2.8)	0.552	8.1 (2.4)	8.5 (3.0)	0.045
HbA1c, mean (SD), %	9.6 (2.3)	9.8 (2.6)	0.589	9.7 (2.4)	10.2 (9.8)	0.053
C-peptide	1.4 (1.0 to 2.0)	1.0 (0.7 to 1.7)	<0.001	1.5 (1.0 to 2.1)	0.9 (0.5 to 1.5)	<0.001
Total cholesterol, mean (SD), mmol/L	5.2 (1.3)	5.2 (1.5)	0.983	5.1 (1.4)	5.0 (1.4)	0.700
HDL cholesterol, mean (SD), mmol/L	1.3 (0.3)	1.3 (0.4)	0.115	1.2 (0.4)	1.2 (0.3)	0.703
LDL cholesterol, mean (SD), mmol/L	3.0 (1.1)	3.2 (1.2)	0.166	2.7 (2.3)	3.1 (1.1)	0.008
ALT, mean (SD), U/L	25.6 (18.2)	17.9 (14.6)	0.001	33.4 (38.4)	28.1 (29.3)	0.087
AST, mean (SD), U/L	22.0 (12.2)	17.5 (7.8)	0.003	24.1 (19.5)	22.8 (20.0)	0.454
Triglycerides, median (IQR), mmol/L	1.6 (1.2 to 2.4)	1.1 (0.9 to 1.7)	<0.001	1.6 (1.1 to 2.6)	1.1 (0.8 to 1.6)	<0.001
Heart failure, n (%)	14 (3.9)	2 (2.6)	0.559	14 (3.6)	11 (4.2)	0.672
Coronary heart disease, n (%)	97 (27.3)	22 (28.2)	0.875	114 (29.2)	92 (35.40	0.094
Diabetic nephropathy, n (%)	89 (25.1)	13 (16.7)	0.113	83 (21.2)	49 (18.9)	0.46
Diabetic retinopathy, n (%)	174 (49.0)	42 (53.9)	0.44	164 (42.0)	125 (48.1)	0.123
Diabetic peripheral neuropathy, n (%)	97 (27.3)	20 (25.6)	0.762	99 (25.3)	87 (33.5)	0.024
Hypoglycemia, n (%)	41 (11.6)	11 (14.1)	0.530	36 (9.2)	39 (15.0)	0.023
MBG, mean (SD), mmol/L	10.2 (1.9)	10.2 (2.0)	0.865	9.9 (1.7)	10.3 (1.7)	0.003
PPGE, mean (SD), mmol/L	2.8 (0.9)	2.9 (0.7)	0.135	2.9 (1.0)	3.2 (1.0)	0.002
LAGE, mean (SD), mmol/L	13.1(3.4)	13.0 (3.7)	0.816	12.9 (3.2)	14.2 (3.1)	<0.001
SDBG, mean (SD), mmol/L	3.0 (0.9)	3.1(1.0)	0.620	3.1 (0.9)	3.4(0.9)	<0.001
Insulin dosage (units per day per kg)	0.73 (0.2)	0.72 (0.3)	0.312	0.73 (0.2)	0.72 (0.3)	0.398

Values are mean (SD), or median [IQR] for continuous variables, and N (%) for categorical variables. BMI, body mass index; MBG, mean blood glucose; PPGE, postprandial glucose excursion; LAGE, large amplitude of glycemic excursions; SDBG, standard deviation of MBG.

We observed a significant and sex-specific interaction between the status of low muscle mass and glucose fluctuations (LAGE and SDBG) (p for interaction=0.025 and 0.036 for SDBG and LAGE, respectively). In **Table 2**, Among males, low muscle mass was significantly associated with a higher LAGE and SDBG (difference in LAGE: 2.26 [95% CI: 1.01 to 3.51], *p* < 0.001; difference in SDBG: 0.45 [95% CI: 0.25 to 0.65], *p* < 0.001) after adjustment for HbA1c, diabetes duration, hyperlipidemia, diabetic peripheral neuropathy, diabetic nephropathy, and cardiovascular disease (Model 1). These associations remained significant after further adjustment for age (difference in LAGE: 2.17 [95% CI: 0.92 to 3.41], *p* < 0.001; difference in SDBG: 0.41 [95% CI: 0.21 to 0.61], *p* < 0.001 in Model 2), and C-peptide (difference in LAGE: 1.18 [95% CI: 0.51 to 3.11], *p* = 0.006; difference in SDBG: 0.31 [95% CI: 0.11 to 0.52], *p* = 0.003 in Model 3) (**Table 2**). However, among females, no significant association between low muscle mass and LAGE, or SDBG was observed after adjustment for all covariates.

**Table 2:** Association of low muscle mass with glucose fluctuations (LAGE and SDBG) among participants with type 2 diabetes receiving CSII therapy.

	Model 1		Model 2		Model 3	
	Estimate β (95%Cl)	*p* value	Estimate β (95%Cl)	*p* value	Estimate β (95%Cl)	*p* value
**Female**						
LAGE	-0.83 (-3.96 to 2.30)	0.603	-1.18 (-4.35 to 1.99)	0.465	-1.37 (-4.74 to 2.00)	0.426
SDBG	-0.08 (-0.52 to 0.36)	0.724	-0.07 (-0.51 to 0.38)	0.775	-0.13 (-0.60 to 0.34)	0.593
**Male**						
**LAGE**	**2.26 (1.01 to 3.51)**	**<0.001**	**2.17 (0.92 to 3.41)**	**<0.001**	**1.18 (0.51 to 3.11)**	**0.006**
**SDBG**	**0.45 (0.25 to 0.65)**	**<0.001**	**0.41 (0.21 to 0.61)**	**<0.001**	**0.31 (0.11 to 0.52)**	**0.003**

Model 1: adjusted for HbA1c, diabetes duration, hyperlipidemia, diabetic peripheral neuropathy, diabetic nephropathy, and cardiovascular disease;

Model 2: adjusted for covariates in Model 1 + age;

Model 3: adjusted for covariates in Model 2+ C-peptide.

Reference: non- Low muscle mass.

P interaction for between the status of low muscle mass and sex of patients on glucose fluctuations for (LAGE and SDBG) (p for interaction=0.021 and 0.029 for SDBG and LAGE, respectively).

CSII, continuous subcutaneous insulin infusion. The bold values indicates the significant associations (P < 0.05).

In the stratified analysis (**Table 3**), the associations between low muscle mass and LAGE and SDBG were not modified by risk factors in both males and females, including age, BMI, diabetes duration, diabetic nephropathy, diabetic peripheral neuropathy, or cardiovascular disease.

**Table 3:** Subgroup analyses of associations of low muscle mass with glucose fluctuations (LAGE and SDBG) among participants with type 2 diabetes receiving CSII therapy.

		Female				Male		
	N	Estimate β (95%Cl)	*p* value	*p*_interaction_	N	Estimate β (95%Cl)	*p* value	*p*_interaction_
LAGE								
Age				0.566				0.160
<65	326	-0.27 (-4.17 to 3.62)	0.890		546	1.07 (0.06 to 2.08)	0.017	
≥65	107	-2.79 (-9.82 to 4.25)	0.438		105	5.09 (-0.70 to 10.90)	0.085	
SDBG								
Age				0.434				0.173
<65	326	0.08 (-0.52 to 0.69)	0.793		546	0.22 (0.03 to 0.41)	0.023	
≥65	107	-0.46(-1.28 to 0.36)	0.270		105	0.83 (0.05 to 1.61)	0.037	
LAGE								
BMI				0.214				0.664
<18.5	39	1.11 (-0.95 to 3.18)	0.291		61	11.29 (-1.06 to 22.98)	0.073	
18.5-23.9	209	-0.445 (-1.62 to 0.73)	0.459		278	-0.25 (-1.01 to 0.51)	0.523	
24.0-27.9	127	-3.96 (-9.27 to 1.35)	0.143		218	5.70 (2.35 to 9.05)	<0.001	
≥28.0	58	-3.99 (-10.10 to 1.70)	0.312		94	2.71 (-3.34 to 8.76)	0.390	
SDBG								
BMI								0.674
<18.5	39	0.30 (-0.09 to 0.69)	0.131	0.188	61	1.62 (0.10 to 3.16)	0.037	
18.5-23.9	209	0.01 (-0.29 to 0.31)	0.960		278	-0.04 (-0.25 to 0.31)	0.709	
24.0-27.9	127	-1.05 (-2.47 to 0.36)	0.146		218	0.62 (0.13 to 1.10)	0.012	
≥28.0	58	-1.01 (-2.65 to 0.69)	0.204		94	0.59 (-0.99 to 2.18)	0.562	
LAGE								
Diabetes duration				0.946				0.441
<5	20	5.84 (4.64 to 7.04)	<0.001		20	4.07 (1.13 to 7.01)	0.007	
5-9.9	280	-1.51 (-5.62 to 2.59)	0.469		485	1.96 (0.32 to 3.59)	0.019	
≥10	133	-1.67 (-8.53 to 5.20)	0.634		146	1.65 (-0.47 to 0.45)	0.141	
SDBG								
Diabetes duration				0.967				0.491
<5	20	1.64 (0.90 to 2.31)	<0.001		20	-2.81 (-2.87 to -2.75)	<0.001	
5-9.9	280	-0.26 (-0.89 to 0.37)	0.419		485	0.36 (0.10 to 0.62)	0.006	
≥10	133	-0.09 (-0.92 to 0.74)	0.824		146	0.33 (-0.04 to 0.71)	0.083	
LAGE								
Diabetic nephropathy				0.483				0.228
No	331	-0.77 (-3.93 to 2.37)	0.628		519	2.15 (0.49 to 3.82)	0.011	
Yes	102	-2.48 (-13.59 to 9.80)	0.661		132	0.22 (-0.91 to 1.45)	0.724	
SDBG								
Diabetic nephropathy				0.340				0.283
No	331	-0.03 (-0.52 to 0.46)	0.906		519	0.35 (0.10 to 0.60)	<0.001	
Yes	102	-0.36 (-1.78 to 1.06)	0.620		132	0.09 (-0.25 to 0.42)	0.615	
LAGE								
Diabetic peripheral neuropathy				0.308				0.562
No	316	-0.89 (-3.85 to 2.07)	0.555		465	0.84(-0.61 to 2.29)	0.257	
Yes	117	-0.76(-1.81 to 1.49)	0.467		186	0.85 (-0.60 to 2.29)	0.253	
SDBG								
Diabetic peripheral neuropathy				0.647				0.820
No	316	0.17 (-0.04 to 0.38)	0.120		465	0.32 (0.08 to 0.56)	0.009	
Yes	117	0.17 (-0.04 to 0.38)	0.116		186	0.33 (-0.04 to 0.08)	0.100	
LAGE								
Cardiovascular disease				0.232				0.567
No	314	-0.09 (-3.67 to 3.50)	0.962		445	1.89 (0.06 to 3.72)	0.043	
Yes	119	-2.25 (-10.46 to 5.95)	0.591		206	1.49 (0.02 to 2.96)	0.046	
SDBG								
Cardiovascular disease				0.153				0.606
No	314	0.10 (-0.46 to 0.65)	0.734		445	0.34 (0.05 to 0.62)	0.019	
Yes	119	-0.45 (-1.42 to 0.53)	0.370		206	0.26 (-0.003 to 0.52)	0.052	

Adjusted for HbA1c, diabetes duration, hyperlipidemia, diabetic peripheral neuropathy, diabetic nephropathy, cardiovascular disease, age, and C-peptide.

CSII, continuous subcutaneous insulin infusion.

Reference: non- Low muscle mass.

## Discussion

In the present study, based on the included 1084 hospitalized patients with T2DM receiving CSII therapy, we found the prevalence of low muscle mass was higher in males than in females and a significant sex-specific association between low muscle mass and glucose fluctuations (LAGE and SDBG). Low muscle mass was significantly associated with a higher LAGE and SDBG for males after adjustment for HbA1c, diabetes duration, hyperlipidemia, diabetic peripheral neuropathy, diabetic nephropathy, cardiovascular disease, age, and C-peptide, but not for females.

Our findings were in line with a previous study that showed that glucose fluctuations were related to low muscle mass. In the study of 69 T2DM patients diagnosed with or without cognitive impairment, glucose fluctuations were found to be independently associated with sarcopenia, even after adjusting for HbA1c levels and associated factors among patients with cognitive impairment (11). However, the study was based on small sample size and whether the association of glucose fluctuations with sarcopenia among diabetes patients with cognitive impairment was modified by sex was unclear. In addition, previous studies have shown that poor glycemic control was associated with poor lower-limb muscle quality, physical performance, and knee extensor strength (18, 19).

To the best of our knowledge, there was no study performed to explore the role of sex-dependent differences in the relationship between low muscle mass and glucose fluctuations. In our study including 1084 hospitalized patients with T2DM receiving CSII therapy, we found that the association between low muscle mass and glucose fluctuations was sex-specific. Low muscle mass was significantly associated with a higher LAGE and SDBG for males after adjustment for HbA1c, diabetes duration, hyperlipidemia, diabetic peripheral neuropathy, diabetic nephropathy, and cardiovascular disease. A significant relationship has been repeatedly reported for association between sarcopenia and age in T2DM individuals (7, 20). Pancreatic β-cell function is an established risk factor for glucose fluctuations (3, 21, 22). To assess the association between low muscle mass and glucose fluctuations, we adjusted age and C-peptide further. Those associations were also significant after adjustment for age and C-peptide. Those associations were also significant after adjustment for age and C-peptide. But among females, low muscle mass was not associated with LAGE or SDBG after adjustment for all covariates. Low muscle mass is the key component of sarcopenia. There are interactions between T2DM and sarcopenia, and the existence of one disease may increase the risk of developing the other (8, 9). T2DM can negatively affect muscle health through insulin resistance (23, 24), advanced glycation end-products (AGEs) accumulation (25), inflammation (26, 27), oxidative stress (25), impaired protein metabolism (19, 28), vascular mitochondrial dysfunction, and cell death (9). In addition, glucose fluctuation is a greater trigger of oxidative stress and inflammation than sustained hyperglycemia (3, 29, 30) and may be involved in the development and progression of low muscle mass. Low muscle mass induced altered glucose disposal (10) and inter and intramuscular adipose tissue accumulation increased local inflammation (31), furthermore, sarcopenia may result in deterioration for the development and progression of T2DM.

In our study, the prevalence of low muscle mass was higher in males than in females. Low muscle mass was significantly associated with a higher LAGE and SDBG for males, but not for females. Previous researchers have revealed a sex gap in metabolic regulation, diabetes susceptibility and risks for sarcopenia amongst community-dwelling older adults, according to which, males were more likely to be diabetes and sarcopenic (32, 33). The underlying mechanism for such sex difference in the association between low muscle mass and fluctuations is unclear, whereas several potential biological mechanisms may contribute. Firstly, sex hormones play diverse roles in maintaining skeletal muscle homeostasis. Testosterone could exert an anabolic effect on skeletal muscle and estrogens have a protective effect on skeletal muscle. Age-induced sex hormone changes contribute to muscle wasting (34). During the aging process, levels of testosterone and insulin-like growth factor-1 could significantly decrease in males that leading to a rapid loss of muscle mass and strength, which significantly increase the risk of sarcopenia (35). As the largest organ responsible for insulin-induced glucose disposal in humans, the rapid loss of the skeletal muscle in males might lead to diminished insulin-induced glucose disposal and exacerbated insulin resistance, resulting in severe glucose abnormalities (36). Secondly, there are sex differences in metabolic adaption and diabetes susceptibility. Males are more likely to develop obesity, insulin resistance, and hyperglycemia than females in response to nutritional challenges (12). Besides, future studies are required to explore how sex differences contribute to the special association between low muscle mass and glucose fluctuations, further investigations could explore other mechanisms.

A major strength of this study is a large sample of hospitalized T2DM patients receiving CSII therapy was included and the monitoring of capillary blood glucose and tailoring of insulin dosage was conducted following the standard protocol by trained doctors and nurses. There are several limitations in our study. Firstly, due to the limitation of observational studies, they could not control factors that might affect the results of the study, and therefore, we could not identify a causal relationship between low muscle mass and glucose fluctuations in males. Secondly, some detailed information, such as physical activity and low muscle strength which may impact glucose control, was not available in this study. Thirdly, standard capillary blood glucose monitoring was applied to evaluate glucose levels, while continuous glucose monitoring (CGM) might represent a more accurate glucose profile. However, the cost of CGM is too high to apply in routine clinical practice in China. Self-monitoring of blood glucose is still commonly used to determine glycemic variability indices, especially in developing countries (37–39). We measured glucose levels 7 times per day by a trained nurse using a unified glucometer on 7 separate days during the in-hospital period, our data could reflect the characteristics of glucose profiles over this period. Finally, major participants were Chinese, further study should be generalized to other populations.

## Conclusion

In the present study based on hospitalized patients with T2DM receiving CSII therapy, we found the prevalence of low muscle mass was higher in males than in females and a significant sex-specific association between low muscle mass and glucose fluctuations (LAGE and SDBG). Low muscle mass was significantly associated with a higher LAGE and SDBG for males, but not for females. The findings suggest that we should pay more attention to glucose fluctuations in male T2DM patients with low muscle mass when using medication to control glucose in clinical practice.

## Data Availability Statement

The raw data supporting the conclusions of this article will be made available by the authors, without undue reservation.

## Ethics Statement

The studies involving human participants were reviewed and approved by The ethics committee of the First Affiliated Hospital of Xiamen University. The patients/participants provided their written informed consent to participate in this study.

## Author Contributions

XLS, WJL, WL, and XJL were involved in the design of the study. XLS conducted the data analysis. All authors were involved in the recruitment of participants and blood sample collection. XLS and WJL completed the first draft of the manuscript. All authors were involved in the critical revision of the manuscript. All authors read and approved the final manuscript. XLS, WJL, WL, and XJL guarantee this work and take responsibility for the integrity of the data. All authors contributed to the article and approved the submitted version.

## Funding

This work was supported by the Natural Science Foundation of Fujian Province, China (No. 2021J011344) and Medical and Health Project of Xiamen (No. 3502Z20214ZD1025).

## Conflict of Interest

The authors declare that the research was conducted in the absence of any commercial or financial relationships that could be construed as a potential conflict of interest.

## Publisher’s Note

All claims expressed in this article are solely those of the authors and do not necessarily represent those of their affiliated organizations, or those of the publisher, the editors and the reviewers. Any product that may be evaluated in this article, or claim that may be made by its manufacturer, is not guaranteed or endorsed by the publisher.

## References

[1] Ceriello A Kilpatrick ES. Glycemic Variability: Both Sides of the Story. Diabetes Care (2013) 36 (Suppl 2):S272–5. doi: 10.2337/dcS13-2030 PMC3920802 23882058

[2] Škrha J Šoupal J Škrha J Jr Prázný M. Glucose Variability, HbA1c and Microvascular Complications. Rev endocr Metab Disord (2016) 17(1):103–10. doi: 10.1007/s11154-016-9347-2 26975588

[3] Zhang ZY Miao LF Qian LL Wang N Qi MM Zhang YM. Molecular Mechanisms of Glucose Fluctuations on Diabetic Complications. Front Endocrinol (2019) 10:640. doi: 10.3389/fendo.2019.00640 PMC6759481 31620092

[4] Cruz-Jentoft AJ Bahat G Bauer J Boirie Y Bruyère O Cederholm T. Sarcopenia: Revised European Consensus on Definition and Diagnosis. Age Ageing (2019) 48(1):16–31. doi: 10.1093/ageing/afy169 30312372 PMC6322506

[5] Anagnostis P Gkekas NK Achilla C Pananastasiou G Taouxidou P Mitsiou M. Type 2 Diabetes Mellitus Is Associated With Increased Risk of Sarcopenia: A Systematic Review and Meta-Analysis. Calcified Tissue Int (2020) 107(5):453–63. doi: 10.1007/s00223-020-00742-y 32772138

[6] Veronese N Stubbs B Punzi L Soysal P Incalzi RA Saller A. Effect of Nutritional Supplementations on Physical Performance and Muscle Strength Parameters in Older People: A Systematic Review and Meta-Analysis. Ageing Res Rev (2019) 51:48–54. doi: 10.1016/j.arr.2019.02.005 30826500

[7] Izzo A Massimino E Riccardi G Della Pepa G. A Narrative Review on Sarcopenia in Type 2 Diabetes Mellitus: Prevalence and Associated Factors. Nutrients (2021) 13(1):183. doi: 10.3390/nu13010183 PMC7826709 33435310

[8] Liccini A Malmstrom TK. Frailty and Sarcopenia as Predictors of Adverse Health Outcomes in Persons With Diabetes Mellitus. J Am Med Directors Assoc (2016) 17(9):846–51. doi: 10.1016/j.jamda.2016.07.007 27569712

[9] Mesinovic J Zengin A De Courten B Ebeling PR Scott D. Sarcopenia and Type 2 Diabetes Mellitus: A Bidirectional Relationship. Diabetes Metab syndrome obesity: Targets Ther (2019) 12:1057–72. doi: 10.2147/dmso.S186600 PMC6630094 31372016

[10] Scott D de Courten B Ebeling PR. Sarcopenia: A Potential Cause and Consequence of Type 2 Diabetes in Australia's Ageing Population? Med J Aust (2016) 205(7):329–33. doi: 10.5694/mja16.00446 27681976

[11] Ogama N Sakurai T Kawashima S Tanikawa T Tokuda H Satake S. Association of Glucose Fluctuations With Sarcopenia in Older Adults With Type 2 Diabetes Mellitus. J Clin Med (2019) 8(3):319. doi: 10.3390/jcm8030319 PMC6463152 30845785

[12] Tramunt B Smati S Grandgeorge N Lenfant F Arnal JF Montagner A. Sex Differences in Metabolic Regulation and Diabetes Susceptibility. Diabetologia (2020) 63(3):453–61. doi: 10.1007/s00125-019-05040-3 PMC6997275 31754750

[13] Zhou B. [Predictive Values of Body Mass Index and Waist Circumference to Risk Factors of Related Diseases in Chinese Adult Population]. Zhonghua liu xing bing xue za zhi = Zhonghua liuxingbingxue zazhi (2002) 23(1):5–10. 12015100

[14] Chen LK Liu LK Woo J Assantachai P Auyeung TW Bahyah KS. Sarcopenia in Asia: Consensus Report of the Asian Working Group for Sarcopenia. J Am Med Directors Assoc (2014) 15(2):95–101. doi: 10.1016/j.jamda.2013.11.025 24461239

[15] Chen L Sun W Liu Y Zhang L Lv Y Wang Q. Association of Early-Phase In-Hospital Glycemic Fluctuation With Mortality in Adult Patients With Coronavirus Disease 2019. Diabetes Care (2021) 44(4):865–73. doi: 10.2337/dc20-0780 33479161

[16] Sokooti S Kieneker LM Borst MH Muller Kobold A Kootstra-Ros JE Gloerich J. Plasma C-Peptide and Risk of Developing Type 2 Diabetes in the General Population. J Clin Med (2020) 9(9):3001. doi: 10.3390/jcm9093001 PMC7564789 32957570

[17] Jeyam A Colhoun H McGurnaghan S Blackbourn L McDonald TJ Palmer CNA. Clinical Impact of Residual C-Peptide Secretion in Type 1 Diabetes on Glycemia and Microvascular Complications. Diabetes Care (2021) 44(2):390–8. doi: 10.2337/dc20-0567 33303639

[18] Yoon JW Ha YC Kim KM Moon JH Choi SH Lim S. Hyperglycemia Is Associated With Impaired Muscle Quality in Older Men With Diabetes: The Korean Longitudinal Study on Health and Aging. Diabetes Metab J (2016) 40(2):140–6. doi: 10.4093/dmj.2016.40.2.140 PMC4853221 27126884

[19] Kalyani RR Metter EJ Egan J Golden SH Ferrucci L. Hyperglycemia Predicts Persistently Lower Muscle Strength With Aging. Diabetes Care (2015) 38(1):82–90. doi: 10.2337/dc14-1166 25392294 PMC4274779

[20] Murata Y Kadoya Y Yamada S Sanke T. Sarcopenia in Elderly Patients With Type 2 Diabetes Mellitus: Prevalence and Related Clinical Factors. Diabetol Int (2018) 9(2):136–42. doi: 10.1007/s13340-017-0339-6 PMC6224944 30603361

[21] Shao C Gu J Meng X Zheng H Wang D. Systematic Investigation Into the Role of Intermittent High Glucose in Pancreatic Beta-Cells. Int J Clin Exp Med (2015) 8(4):5462–9. PMC4483841 26131124

[22] Murata M Adachi H Oshima S Kurabayashi M. Glucose Fluctuation and the Resultant Endothelial Injury are Correlated With Pancreatic β Cell Dysfunction in Patients With Coronary Artery Disease. Diabetes Res Clin Pract (2017) 131:107–15. doi: 10.1016/j.diabres.2017.07.007 28743060

[23] Kuo CK Lin LY Yu YH Wu KH Kuo HK. Inverse Association Between Insulin Resistance and Gait Speed in Nondiabetic Older Men: Results From the U.S. National Health and Nutrition Examination Survey (NHANES) 1999-2002. BMC geriatrics (2009) 9:49. doi: 10.1186/1471-2318-9-49 19922671 PMC2784762

[24] Lee CG Boyko EJ Strotmeyer ES Lewis CE Cawthon PM Hoffman AR. Association Between Insulin Resistance and Lean Mass Loss and Fat Mass Gain in Older Men Without Diabetes Mellitus. J Am Geriatrics Soc (2011) 59(7):1217–24. doi: 10.1111/j.1532-5415.2011.03472.x PMC3716256 21718263

[25] Aragno M Mastrocola R Catalano MG Brignardello E Danni O Boccuzzi G. Oxidative Stress Impairs Skeletal Muscle Repair in Diabetic Rats. Diabetes (2004) 53(4):1082–8. doi: 10.2337/diabetes.53.4.1082 15047625

[26] Park SW Goodpaster BH Strotmeyer ES Kuller LH Broudeau R Kammerer C. Accelerated Loss of Skeletal Muscle Strength in Older Adults With Type 2 Diabetes: The Health, Aging, and Body Composition Study. Diabetes Care (2007) 30(6):1507–12. doi: 10.2337/dc06-2537 17363749

[27] Payette H Roubenoff R Jacques PF Dinarello CA Wilson PW Abad LW. Insulin-Like Growth Factor-1 and Interleukin 6 Predict Sarcopenia in Very Old Community-Living Men and Women: The Framingham Heart Study. J Am Geriatrics Soc (2003) 51(9):1237–43. doi: 10.1046/j.1532-5415.2003.51407.x 12919235

[28] Russell ST Rajani S Dhadda RS Tisdale MJ. Mechanism of Induction of Muscle Protein Loss by Hyperglycaemia. Exp Cell Res (2009) 315(1):16–25. doi: 10.1016/j.yexcr.2008.10.002 18973755

[29] Ceriello A Esposito K Piconi L Ihnat MA Thorpe JE Testa R. Oscillating Glucose Is More Deleterious to Endothelial Function and Oxidative Stress Than Mean Glucose in Normal and Type 2 Diabetic Patients. Diabetes (2008) 57(5):1349–54. doi: 10.2337/db08-0063 18299315

[30] Ohara M Nagaike H Goto S Fukase A Tanabe Y Tomoyasu M. Improvements of Ambient Hyperglycemia and Glycemic Variability Are Associated With Reduction in Oxidative Stress for Patients With Type 2 Diabetes. Diabetes Res Clin Pract (2018) 139:253–61. doi: 10.1016/j.diabres.2018.02.017 29501829

[31] Marcus RL Addison O Dibble LE Foreman KB Morrell G Lastayo P. Intramuscular Adipose Tissue, Sarcopenia, and Mobility Function in Older Individuals. J Aging Res (2012) 2012:629637. doi: 10.1155/2012/629637 22500231 PMC3303569

[32] Tay L Ding YY Leung BP Ismail NH Yeo A Yew S. Sex-Specific Differences in Risk Factors for Sarcopenia Amongst Community-Dwelling Older Adults. Age (Dordrecht Netherlands) (2015) 37(6):121. doi: 10.1007/s11357-015-9860-3 PMC5005859 26607157

[33] Du Y Wang X Xie H Zheng S Wu X Zhu X. Sex Differences in the Prevalence and Adverse Outcomes of Sarcopenia and Sarcopenic Obesity in Community Dwelling Elderly in East China Using the AWGS Criteria. BMC endocr Disord (2019) 19(1):109. doi: 10.1186/s12902-019-0432-x 31653213 PMC6814981

[34] Anderson LJ Liu H Garcia JM. Sex Differences in Muscle Wasting. Adv Exp Med Biol (2017) 1043:153–97. doi: 10.1007/978-3-319-70178-3_9 29224095

[35] Kim KM Jang HC Lim S. Differences Among Skeletal Muscle Mass Indices Derived From Height-, Weight-, and Body Mass Index-Adjusted Models in Assessing Sarcopenia. Korean J Internal Med (2016) 31(4):643–50. doi: 10.3904/kjim.2016.015 PMC4939509 27334763

[36] NCD Risk Factor Collaboration (NCD-RisC) Abarca-Gómez L Abdeen ZA Abdul Hamid Z Abu-Rmeileh NM Acosta-Cazares B Acuin C. Worldwide Trends in Body-Mass Index, Underweight, Overweight, and Obesity From 1975 to 2016: A Pooled Analysis of 2416 Population-Based Measurement Studies in 128.9 Million Children, Adolescents, and Adults. Lancet (London England) (2017) 390(10113):2627–42. doi: 10.1016/s0140-6736(17)32129-3 PMC5735219 29029897

[37] Klatman EL Jenkins AJ Ahmedani MY Ogle GD. Blood Glucose Meters and Test Strips: Global Market and Challenges to Access in Low-Resource Settings. Lancet Diabetes Endocrinol (2019) 7(2):150–60. doi: 10.1016/s2213-8587(18)30074-3 30072234

[38] Weinstock RS Braffett BH McGuigan P Larkin ME Grover NB Walders-Abramson N. Self-Monitoring of Blood Glucose in Youth-Onset Type 2 Diabetes: Results From the TODAY Study. Diabetes Care (2019) 42(5):903–9. doi: 10.2337/dc18-1854 PMC6489117 30833375

[39] Zhang Y Dai J Han X Zhao Y Zhang H Liu X. Glycemic Variability Indices Determined by Self-Monitoring of Blood Glucose Are Associated With β-Cell Function in Chinese Patients With Type 2 Diabetes. Diabetes Res Clin Pract (2020) 164:108152. doi: 10.1016/j.diabres.2020.108152 32360707

